# Getting to the Root of Fine Motor Skill Performance in Dentistry: Brain Activity During Dental Tasks in a Virtual Reality Haptic Simulation

**DOI:** 10.2196/jmir.8046

**Published:** 2017-12-12

**Authors:** Suzanne Perry, Susan M Bridges, Frank Zhu, W Keung Leung, Michael F Burrow, Jamie Poolton, Rich SW Masters

**Affiliations:** ^1^ Faculty of Education The University of Hong Kong Hong Kong SAR China; ^2^ Centre for the Enhancement of Teaching and Learning The University of Hong Kong Hong Kong SAR China; ^3^ Department of Surgery Li Ka Shing Faculty of Medicine The University of Hong Kong Hong Kong SAR China; ^4^ Faculty of Dentistry The University of Hong Kong Hong Kong SAR China; ^5^ Melbourne Dental School The University of Melbourne Melbourne Australia; ^6^ Carnegie School of Sport Leeds Beckett University Leeds United Kingdom; ^7^ School of Public Health Li Ka Shing Faculty of Medicine The University of Hong Kong Hong Kong SAR China; ^8^ Te Huataki Waiora Faculty of Health, Sport and Human Performance The University of Waikato Hamilton New Zealand

**Keywords:** simulation, fNIRS, functional near-infrared spectroscopy, spectroscopy, near-infrared, virtual reality, psychomotor skills training, dentistry, education, medical

## Abstract

**Background:**

There is little evidence considering the relationship between movement-specific reinvestment (a dimension of personality which refers to the propensity for individuals to consciously monitor and control their movements) and working memory during motor skill performance. Functional near-infrared spectroscopy (fNIRS) measuring oxyhemoglobin demands in the frontal cortex during performance of virtual reality (VR) psychomotor tasks can be used to examine this research gap.

**Objective:**

The aim of this study was to determine the potential relationship between the propensity to reinvest and blood flow to the dorsolateral prefrontal cortices of the brain. A secondary aim was to determine the propensity to reinvest and performance during 2 dental tasks carried out using haptic VR simulators.

**Methods:**

We used fNIRS to assess oxygen demands in 24 undergraduate dental students during 2 dental tasks (clinical, nonclinical) on a VR haptic simulator. We used the Movement-Specific Reinvestment Scale questionnaire to assess the students’ propensity to reinvest.

**Results:**

Students with a high propensity for movement-specific reinvestment displayed significantly greater oxyhemoglobin demands in an area associated with working memory during the nonclinical task (Spearman correlation, *r*_s_=.49, *P*=.03).

**Conclusions:**

This small-scale study suggests that neurophysiological differences are evident between high and low reinvesters during a dental VR task in terms of oxyhemoglobin demands in an area associated with working memory.

## Introduction

One consequence of working-time directives and curriculum reform in recent years has been a reduction in health care training hours, with claims of up to a 5-fold cut in surgical training hours in some medical specialties [1,2]. In undergraduate dentistry, supervised curriculum hours dedicated to psychomotor skills training have been reduced as a consequence of overcrowding of dental curricula with new material [3]. Inevitably, removal of some of the more hands-on practical components of dental curricula [4] is likely to reduce the amount of psychomotor skills teaching time that undergraduates experience.

As a result, there has been considerable interest among health care professions in identification of individuals who may struggle to acquire the required standard of psychomotor skills by the end of their training [5]. In dentistry, regardless of their progress, all students receive the same number of hours of training. Costly, time-consuming classes are required for individuals who do not achieve the set standards [6]. In response, research has been conducted to establish factors that predict psychomotor skills performance [5,7]. Consistent with other high-risk professions, such as aviation [8], in dentistry personality is increasingly being identified as an important factor in health care psychomotor performance [9].

Surveys of surgeons have identified particular components of personality that are important for general performance [2,5], with conscientiousness having been shown to be a predictor of success across the entire medical undergraduate curriculum [10,11]. Conscientiousness has also been implicated in successful psychomotor performance in dentistry [9,12]. Other aspects of personality thought to play a role are introversion in surgery [11], and warmth, agreeableness, trust, straightforwardness, and compliance in dental technology [9]. The mechanisms underlying the influence of such personality traits are not well understood, however.

One dimension of personality that may have a more direct effect on surgical learning and performance is movement-specific reinvestment, which refers to the propensity for individuals to consciously monitor and control their movements [13,14]. A 10-item questionnaire, the Movement-Specific Reinvestment Scale (MSRS) [15], has been developed and validated to measure this trait. Recent empirical work has shown that the propensity for an individual to consciously monitor and control their movements affects skill acquisition. For example, MSRS scores have been shown to predict the rate of acquisition of a simple laparoscopic skill [16,17].

Studies have shown that greater demands are placed on working memory in movement contexts that induce conscious monitoring and control than in those in which movement control is more automated [18,19]. Working memory is a brain system that is involved in active maintenance, manipulation, and storage of information related to current tasks [20]. Conscious monitoring and control thus depends on working memory [13], so people who score high on the MSRS seem to be more reliant on working memory than those who score low on the MSRS [21,22].

Most research on movement-specific reinvestment has focused on performance outcomes, but little research has examined evidence at a neurological level. One exception is empirical work by Zhu et al [23], who used electroencephalographic measures of corticocortical communication during a golf putting task to demonstrate that people who had a high propensity for reinvestment tended to have greater coactivation between verbal-analytical areas of the brain and motor planning region than did people with a low propensity for reinvestment [24]. The authors concluded that their data presented objective neural evidence that movement-specific reinvestment represents the extent to which an individual is consciously engaged in the process of moving.

In related work, examining neural activity during surgery, Ohuchida et al [25] used functional near-infrared spectroscopy (fNIRS) to demonstrate that trainees with no experience of an endoscopy task had higher oxyhemoglobin demands in the frontal cortex of the brain than expert surgeons. fNIRS is a technique that uses a series of near-infrared light sources and detectors across the surface of the scalp to measure levels of oxygenated and deoxygenated blood in specific regions of the brain. A typical hemodynamic response to functional brain activation involves an increase in oxyhemoglobin and a decrease in deoxyhemoglobin during a task when compared with rest. One study has suggested that oxyhemoglobin responses in the prefrontal regions of the brain are associated with working memory activity [26].

In particular, the dorsolateral prefrontal cortex (DLPFC) is an area of the brain that is heavily involved in executive functions associated with working memory and motor planning [27]. The left DLPFC appears to be involved in observation of new information, preparation for movement, and the creation of new motor patterns, whereas the right DLPFC is implicated in effort-demanding tasks and the supervision and monitoring of movement [28]. What is not yet understood is the relationship between a person’s propensity to consciously monitor and control their movements and oxyhemoglobin demands in the DLPFC when performing a dental procedural task. We expected a positive relationship. That is, individuals with a high propensity for movement-specific reinvestment should exhibit greater oxyhemoglobin demands in the DLPFC, reflecting higher levels of working memory engagement in performance.

The aim of this fNIRS study was to investigate whether propensity for movement-specific reinvestment was associated with oxygen demands in the DLPFC during completion of simple and complex dental haptic virtual reality (VR) simulator tasks. By understanding such differences at the neurological level in fine-motor dental tasks, educators can begin to develop training interventions that are better tailored to the needs of individual learners.

## Methods

Ethical approval of the study was granted by the University of Hong Kong ethics research board and written informed consent was gained from all participants prior to taking part.

### Participants

We recruited volunteer fourth-year dental students who had accumulated 6 hours of dedicated curriculum time on haptic VR simulators during the first year of their dental undergraduate degree course, 3 years prior to the start of this study. Between the end of their first year and the start of the study, no additional formal haptic VR simulator sessions were delivered. The students all had real-life caries management experience (beginning midway through their second year), consisting of approximately 3 half days a week of general dentistry by the end of their third year. Records from the haptic hard drive indicated that none of the students had used the haptic VR simulators in their free time in the 3-year period between the haptic VR simulator training in first year and the start of this study.

### Dental Haptic Virtual Reality Simulator

**Figure 1:**
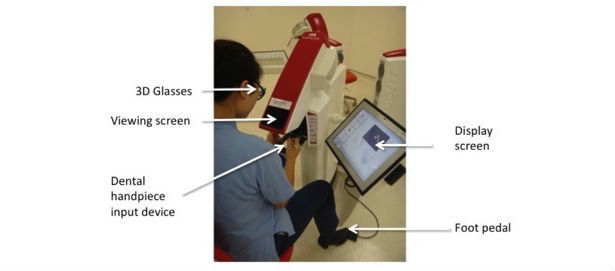
The Simodont haptic dental simulator. 3D: 3-dimensional.

The tasks were carried out on the Simodont dental haptic VR simulator (MOOG Inc, Buffalo, NY, USA). With the use of 3-dimensional (3D) glasses, this simulator unit allows visualization of a projected image within a viewing screen, which can be modified using an input device similar to a dental handpiece (drill). Haptic sensory feedback is incorporated into the “handpiece,” providing a more realistic simulation. In addition to the viewing screen, a second screen displays output performance data and a foot control allows the speed of the handpiece to be adjusted, thereby simulating real-life performance (Figure 1).

### Functional Near-Infrared Spectroscopy

**Figure 2:**
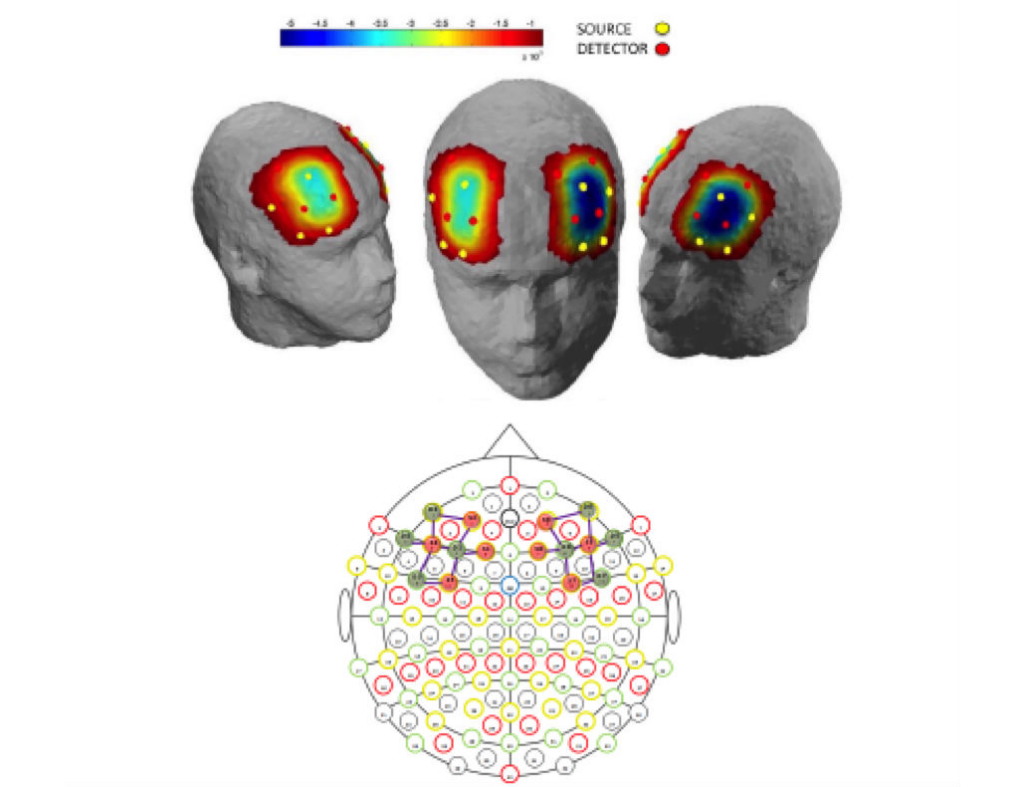
Locations of sources, detectors, and channels and underlying graphic representation of oxyhemoglobin concentration in the dorsolateral prefrontal cortex during a dental virtual reality task.

We used a multichannel portable fNIRS system, NIRSport (NIRx Medical Technologies LLC, Glen Head, NY, USA), to detect the attenuated dual wavelength signals (760 nm and 850 nm) from the left and right DLPFC. We took head measurements to allow accurate placement of the fNIRS cap bilaterally over the prefrontal region. The cap contained 8 source and 8 detector LEDs, allowing blood oxygenation levels to be recorded from 18 channels with a sampling rate of 7.81 Hz (Figure 2).

We set the distance between sources and detectors at approximately 3 cm, with the arrangement of the channels compatible with that of the international 10-5 system. Data from the detectors were transferred directly to a laptop computer, with a second laptop computer synchronizing the verbal cues “rest,” “start,” “rest,” and “done” with the marking of recorded data. Video recordings were taken of all participants during the data collection period to allow observations to be made retrospectively. Screen capture software (Snagit, TechSmith Corporation) on the central network computer recorded completion of the tasks at the 3D image level.

### Procedure

**Figure 3:**
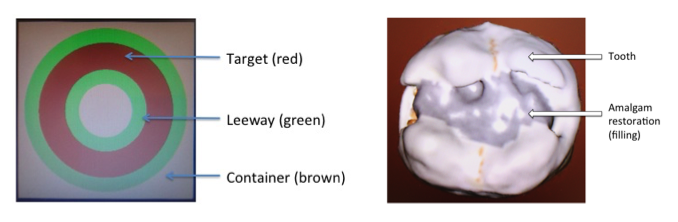
Circle (left) and tooth (right) tasks using the virtual reality simulator.

Participants completed 2 tasks (circle, tooth) using the VR simulator, the sequencing of which was counterbalanced. The tasks consisted of (1) removing a target area from a 3D circle shape (Figure 3), and (2) removing a silver amalgam restoration (filling) and adjacent caries (decay) from a simulated 3D tooth (Figure 3).

The circle task involved a relatively simple 3D circle shape (Figure 3, left) and had been previously completed by the students as part of their undergraduate haptic psychomotor skills training course. The task required participants to remove as much of the red target band as possible, without damaging the adjacent green leeway areas. Extensive damage to the leeway resulted in damage to the brown container, which was increasingly distant from the target. This task was considered to be simple due to the regularity of the contours and the bold colors clearly defining the regions to be removed and avoided.

The tooth task involved a more complex 3D tooth (Figure 3, right) and had not been completed previously by the students. The task consisted of a simulated tooth containing a 3-surface amalgam restoration (large silver amalgam filling) with adjacent caries (decay). The students were instructed to remove as much of the amalgam and caries as possible without damaging healthy tooth tissue. The task was considered to be more complex due to its similarities to a natural tooth, with irregular and inconsistent features.

Participants were allowed 5 minutes (300 seconds) to carry out each task. They rested for 45 seconds before and after the task to provide a baseline reference for DLPFC activation during the task. During the rest periods, the participants were asked to keep their head as stable as possible to minimize measurement artifacts and to position themselves so that they would be ready to start the task on cue. For each test, the cues (verbal) were produced using voice technology software (NIRStar, NIRx Medical Technologies LLC), with synchronous marking of data and cues to clearly identify task and rest periods for data analysis. Participants were instructed during the tasks to “complete as much of the task as you can, but as carefully as possible.”

### Performance Measures

For the circle task, the simulator automatically recorded the percentage of target and leeway that was removed, and the extent of any damage to the container as a percentage. The resultant scores were defined as target, leeway, and container scores.

**Table 1:** Movement-Specific Reinvestment Scale items.

Item no.	Question
1.	I remember the times when my movements have failed me.
2.	If I see my reflection in a shop window, I examine my movements.
3.	I reflect about my movements a lot.
4.	I try to think about my movements when I carry them out.
5.	I am self-conscious about the way I look when I am moving.
6.	I sometimes have the feeling that I am watching myself move.
7.	I am aware of the way my body moves when I am carrying out a movement.
8.	I am concerned about my style of moving.
9.	I try to figure out why my actions failed.
10.	I am concerned about what people think of me when I am moving.

The tooth task was scored by 5 qualified dentists (mean experience 15.6 years) who blind rated posttask images of the tooth compared with an ideal preparation. We asked the dentists to independently rank the images in relation to 2 specific components: (1) proficiency in removal of the silver amalgam restoration (filling) and caries, and (2) preservation of sound tooth tissue.

Cronbach alpha of .734 suggested that interrater reliability was within an acceptable range (.7-.95) [29]. We then used the mean score of the raters as the dependent value for the tooth task for each participant (the tooth score).

### Functional Near-Infrared Spectroscopy Data Processing and Analysis

We analyzed the fNIRS data using NIRSlab software v2014.05 (NIRx Medical Technologies LLC). The analysis process began with removal of any discontinuities from the data prior to setting a low-pass frequency filter to remove physiological artifacts (0.2 Hz cutoff frequency). We then transformed changes in attenuation at the 760 nm and 850 nm wavelengths into oxygenated and deoxygenated hemoglobin concentration levels, respectively, using the modified Beer-Lambert approach. Overall averaged time-response curves for oxyhemoglobin and deoxyhemoglobin were then plotted for each participant for all 4 tests, allowing a general assessment of data quality. Data from the posttest rest period was more erratic than data from the pretest rest period. Reference to video analysis showed an increase in head movement during the posttest rest period. As a result, we used only the more reliable pretest rest period as a baseline for comparison with the performance test data.

We calculated average levels of oxyhemoglobin and deoxyhemoglobin for the pretest rest and test period of each task. The difference in oxyhemoglobin between rest and test was then calculated to indicate the strength of the hemodynamic response.

### Movement-Specific Reinvestment

We asked all participants to complete the MSRS before they carried out the study tasks. We asked the participants to rate their level of agreement with the 10 statements of the MSRS (Table 1), by indicating on a 6-point Likert scale from “strongly disagree” to “strongly agree.” Scores can range from 10 to 60.

### Statistical Analysis

Due to the relatively low number of participants (n=19), we conducted nonparametric analyses. We used Wilcoxon signed rank tests to determine whether there was a significant effect of task complexity on oxyhemoglobin responses in the DLPFC during the circle (simple) and tooth (complex) tasks. We also used Wilcoxon signed rank tests to examine whether there were differences in oxyhemoglobin and deoxyhemoglobin responses between the left and the right hemisphere of the DLPFC during the tasks.

We used Spearman rank correlation to determine whether an association was present between MSRS scores and oxyhemoglobin and deoxyhemoglobin responses in the left and right DLPFC during the tasks. We also used Spearman rank correlation to determine whether there was an association between MSRS scores and performance of each task, as well as performance of each task and oxyhemoglobin and deoxyhemoglobin demands. We used Fisher *r* to *z* transformations to test for interactions between correlation coefficients. The statistical significance threshold was set to *P*<.05. All statistical analyses were performed using IBM SPSS Statistics for Windows (version 23; IBM Corporation).

## Results

A total of 24 students (13 female, 11 male; mean age 21.5 years, SD 0.52; all right handed) completed the MSRS. Of the 48 trials carried out, 3 trials had an overall negative oxyhemoglobin difference, indicating either an increased response during the rest period or reduced oxyhemoglobin response during the test. In line with other fNIRS studies [30,31], this overall negative response pattern was seen as inconsistent with a hemodynamic response to functional activation. Consequently, we excluded from the study any participant who performed a test with an overall negative oxyhemoglobin response, resulting in the loss of 3 participants. We excluded an additional 2 participants due to equipment failure, resulting in the data from 19 participants (8 high and 11 low reinvesters) being analyzed for this study.

For the deoxyhemoglobin data, no trends or statistical effects were evident. All further data analysis relates to oxyhemoglobin data.

### Oxyhemoglobin Demands and Task Difficulty

The mean overall change in oxyhemoglobin between rest and task completion was 1.22×10^–3^mM in the circle task (simple task) and 1.12×10^–3^mM in the tooth task (complex task). Wilcoxon signed rank test suggested that there was no significant difference in the overall oxyhemoglobin demands between the tasks (*z*=–0.765, *P*=.45).

### Oxyhemoglobin Demands in the Left and Right DLPFC

Table 2 demonstrates the mean overall change in oxyhemoglobin between rest and task for the circle and tooth tasks in the left and right DLPFC. Results indicate a significant difference in the overall oxyhemoglobin demands between the 2 hemispheres during both tasks, with the left hemisphere having more oxyhemoglobin demands.

### Oxyhemoglobin Demands and Movement-Specific Reinvestment

Scoring from the MSRS ranges from 0 to 60. The mean score for reinvestment for the participants in this study was 39.7, SD 5.8. The mean score for the high reinvesters was 44.7, SD 3.9, and that for low reinvesters was 35.5, SD 3.5.

**Figure 4:**
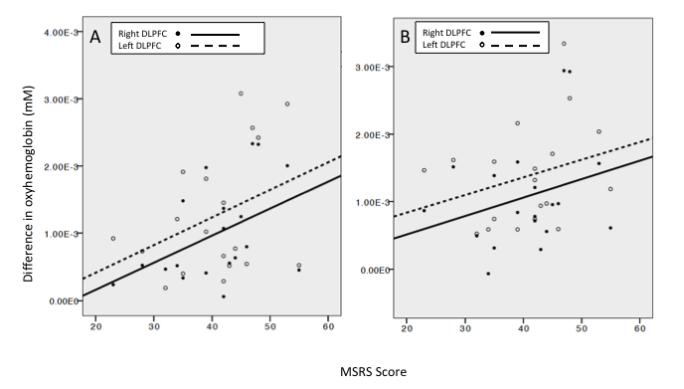
Relationship between score on the Movement-Specific Reinvestment Scale (MSRS) and oxyhemoglobin demand in the left and right dorsolateral prefrontal cortex (DLPFC) during the (A) circle task and (B) tooth task.

Figure 4 shows the association between scores on the MSRS and change in oxyhemoglobin demands in the left and right DLPFC during the circle task and the tooth task, respectively. For both tasks, oxyhemoglobin demands appeared to increase as a function of higher scores on the scale. For the circle task (Figure 4, panel A), Spearman signed rank correlation revealed a significant association between MSRS scores and oxyhemoglobin demands in the right DLPFC (*r*_s_=.49, *P*=.03) but not the left DLPFC (*r*_s_=.30, *P*=.49). Fisher r to z transformation showed that there was no interaction between the left and right DLPFC with respect to score on the MSRS (*z*=–0.66, *P*=.51). For the tooth task (Figure 4, panel B), Spearman signed rank correlation did not reveal significant associations between MSRS scores and oxyhemoglobin demands in the right DLPFC (*r*_s_=.28, *P*=.25) or the left DLPFC (*r*_s_=.35, *P*=.14). Fisher r to z transformation showed that there was no interaction between the left and right DLPFC with respect to score on the MSRS (*z*=–0.21, *P*=.83).

**Table 2:** Mean overall changes in oxyhemoglobin concentration during the tasks by dorsolateral prefrontal cortex hemisphere.

Task	Oxyhemoglobin (mM) by hemisphere		Wilcoxon signed rank test	
	Left	Right	*z*	*P* value
Circle	1.26×10^–3^	9.8×10^–4^	–2.052	.04
Tooth	1.38×10^–3^	1.08×10^–3^	–2.897	.004

### Performance and Oxyhemoglobin Demands

Spearman signed rank tests indicated that there were no significant associations between oxyhemoglobin demands and performance of the circle task (target: *r*_s_=.16, *P*=.94; average leeway: *r*_s_=.10, *P*=.61; and average container: *r*_s_=.25, *P*=.21) or the tooth task (tooth score: *r*_s_=.29, *P*=.19).

### Performance and Movement-Specific Reinvestment

Spearman signed rank tests indicated that there were no significant associations between score on the MSRS and performance of the circle (target, average leeway, and average container removed) or the tooth task (tooth score: *r*_s_ range .060 to .264; target: *P*=.78; average leeway: *P*=.12; average container: *P*=.12).

## Discussion

### Principal Findings

Oxyhemoglobin demands were significantly higher in the left DLPFC during both tasks. Evidence suggests that the left DLPFC has a role in observing new information, preparing for movement, and creating new motor patterns [28]. As both the circle and tooth tasks required movement planning, this may have resulted in significant activation of the left DLPFC.

Interestingly, the hemodynamic response in the DLPFC was not significantly different as a function of task complexity, as has been demonstrated in previous brain imaging studies of the DLPFC [32,33]. The fNIRS literature suggests that there is an increased hemodynamic response in the sensorimotor and visual motion areas as motor task complexity increases [34], and a similar response appears to occur in the motor cortex [35]. However, our data are, to our knowledge, the first to suggest that this may not be the case for the prefrontal cortex.

A higher propensity to consciously control movements, as measured by the MSRS, was associated with increased blood oxyhemoglobin demands in the DLPFC during performance of both tasks, although the association reached significance only in the circle task (see Figure 4, panel A). Nevertheless, neurological differences may exist between high and low reinvesters in the DLPFC during psychomotor dental tasks. This finding is consistent with work by Zhu et al [23], which showed neural differences on electroencephalography between high and low reinvesters during a golf putting task. When the left and right DLPFC were analyzed separately, the association was significant only in the right DLPFC during the circle task. A considerable body of evidence suggests that high reinvesters call upon higher levels of activation of working memory to process and manipulate information during motor tasks [21,22], which is likely to result in increased blood oxyhemoglobin demands [27]. The right DLPFC may be more involved in working memory activities during movement. Fan et al [36] suggested that tasks involving continuous performance and vigilance activate the frontal and parietal regions of the right hemisphere. In music, it has been suggested that the main role of the right DLPFC is to supervise and monitor movement, ensuring that movements match the intended motor pattern [28]. The right DLPFC may well have been increasingly active in monitoring the previous motor pattern formed for the circle task, accounting for the increase in oxyhemoglobin to the region.

Increased effort is linked to resource demands in the right hemisphere [36-38], with tasks of sustained attention or vigilance, in particular, causing an increase in blood flow to the right prefrontal cortex [37]. The circle task had been used as a test task during the students’ previous undergraduate course training. Consequently, students may have had greater expectations of themselves and hence used more effort and attention than in the tooth task. Moreover, the circle task, but not the tooth task, provides an objective score of performance. A monitor linked to the VR haptic simulator shows in real time the percentage of target, leeway, and container that has been removed, which may well have influenced students’ expectations.

Factors such as this may explain the findings, as may other factors such as anxiety, which can increase the chances of movement-specific reinvestment [13,16]. Evidence from other domains suggests that a propensity to reinvest is associated with the likelihood of performance breakdown under pressure and reduced ability to multitask [13,39,40]. Such effects appear to transfer to health care professions as demonstrated in a surgical study in which high reinvesters were less able to cope with time pressure demands [17].

Increasingly, health care professionals are becoming aware of the need for individualized training pathways to improve effectiveness. Evidence from this study points to the possibility of using the MSRS to identify reinvestment characteristics in dental students and to modify training appropriately to reduce the possibility of movement-specific reinvestment [13,39]. Implicit motor learning theory [18,39] argues that this can be done simply by ensuring that an individual learns the motor task without acquiring conscious knowledge of how he or she performs the task. The extent to which implicit motor learning is feasible when learning dental procedures is unclear, but it may have a place during the early stages of dental motor skill training. Structured training when learning dental handpiece skills, for example, could initially be replaced by practice protocols designed to reduce errors, which has been shown to cause implicit motor learning [18]. Even if initial skill training has already taken place, and the opportunity for foundational implicit motor learning has been lost, simple changes, such as focusing on external factors, acclimatization, or even “ritualized” behaviors, may go some way to prevent the effects of reinvestment [13].

Our findings suggest that neurological differences between high and low reinvesters do potentially exist in the working memory region during dental tasks, but it is important to remember that the DLPFC is used for many other brain processes and that, as with many brain imaging techniques, it is difficult to localize the exact regions of the brain that are responding. Additionally, our sample size was small, and it was impossible to quantify each participant’s exact amount of prior experience with each dental task. Further studies addressing these issues would provide invaluable evidence concerning the neurological and psychological processes underpinning dental procedures performed on a haptic VR simulator.

### Conclusion

Although additional studies are necessary to gain further insight into the neurological processes occurring during movement-specific reinvestment, this small study presents neurological evidence suggesting that a higher propensity for conscious control of movements during dental procedures is associated with greater blood oxyhemoglobin demands in the DLPFC, an area associated with working memory activity. The findings provide further support for the theory of reinvestment [13].

As time for psychomotor skill training in health care becomes even more challenged and an increasingly more complex range of surgical skills is required in dentistry [41,42], it is likely that screening of not only innate technical abilities but also personality traits will increase. Screening will potentially allow the identification of individuals who may struggle to achieve predefined psychomotor skill levels in the allotted curriculum time, allowing individualized training pathways to be put in place earlier in the curriculum for such students. Evidence from reinvestment studies suggests that the MSRS may be a useful additional tool for this process.

## Abbreviations

DLPFC: dorsolateral prefrontal cortex
fNIRS: functional near-infrared spectroscopy
MSRS: Movement-Specific Reinvestment Scale
3D: 3-dimensional
VR: virtual reality
